# Author Correction: Direct energy transfer from photosystem II to photosystem I confers winter sustainability in Scots Pine

**DOI:** 10.1038/s41467-021-22013-6

**Published:** 2021-03-08

**Authors:** Pushan Bag, Volha Chukhutsina, Zishan Zhang, Suman Paul, Alexander G. Ivanov, Tatyana Shutova, Roberta Croce, Alfred R. Holzwarth, Stefan Jansson

**Affiliations:** 1 Umeå Plant Science Centre, Department of Plant Physiology, Umeå University, Umeå, Sweden; 2 Department of Physics and Astronomy, Faculty of Sciences, Vrije Universiteit Amsterdam, Amsterdam, The Netherlands; 3 Department of Biology, University of Western Ontario, London, Ontario Canada; 4 Institute of Biophysics and Biomedical Engineering, Bulgarian Academy of Sciences, Sofia, Bulgaria; 5 Present Address: Department of Life Sciences, Imperial College London, London, UK; 6 Present Address: State Key Laboratory of Crop Biology, College of Life Sciences, Shandong Agricultural University, Shandong, China; 7 Present Address: Department of Biochemistry and Biophysics, Stockholm University, Stockholm, Sweden

**Keywords:** Biophysics, Forest ecology, Plant sciences, Non-photochemical quenching

Correction to: *Nature Communications* 10.1038/s41467-020-20137-9, published online 15 December 2020.

The original version of this Article contained an error in Fig. 1g, h and l, in which the *y* axes were labelled ‘NPQ (1-Fm'/Fm)’. They should have been labelled ‘NPQ (Fm/Fm'-1)’. This has been corrected in the PDF and HTML versions of the Article.

