# Supplementary material for: Direct energy transfer from photosystem II to photosystem I confers winter sustainability in Scots Pine
Source: Nat Commun. 2020 Dec 15;11:6388. doi: 10.1038/s41467-020-20137-9 (PMC7738668; doi:10.1038/s41467-020-20137-9)
Supplement: Supplementary file 1 — Supplementary Information [file 41467_2020_20137_MOESM1_ESM.pdf]

Supplementary figures and tables:

Direct energy transfer from photosystem II to photosystem I confers winter sustainability in Scots Pine

Authors

Pushan Bag[1¶], Volha Chukhutsina[2¶≠], Zishan Zhang[1&], Suman Paul[1#], Alexander G. Ivanov[3,4], Tatyana Shutova[1], Roberta Croce[2], Alfred R. Holzwarth[2*], Stefan Jansson[1**]

Affiliations

[1]Umeå Plant Science Centre, Department of Plant Physiology, Umeå University, Umeå, Sweden

[2]Department of Physics and Astronomy, Faculty of Sciences, Vrije Universiteit Amsterdam, Amsterdam, The Netherlands

[3]Department of Biology, University of Western Ontario, London, Ontario, Canada.

[4]Institute of Biophysics and Biomedical Engineering, Bulgarian Academy of Sciences, Sofia, Bulgaria.

[¶] P.B and V.C. contributed equally to this work

[≠] New address: Department of Life Sciences, Imperial College London, United Kingdom

[#] New address: Department of Biochemistry and Biophysics, Stockholm University, Sweden

[&] New address: State Key Laboratory of Crop Biology, College of Life Sciences, Shandong Agricultural University, China

[*] C**orresponding author**: a.holzwarth@vu.nl, stefan.jansson@umu.se

This supplementary file includes:

Supplementary figures 1, 2, 3, 4, 5, 6, 7, 8 and 9 and supplementary table 1, 2, 3 and 4.

Other data file:

Other data sets are provided as source data file which contains all source and raw data for main manuscript figures, supplementary figures, and tables as per the data availability statement provided in the main manuscript.

Supplementary figure 1.

Supplementary figure 1a.

Season 2015-2016

Year	Month	Week
2015	Sept	37
2015	Oct	40
2015	Oct	42
2015	Nov	45
2015	Nov	47
2015	Dec	50
2015	Dec	52
2016	Jan	1
2016	Jan	2
2016	Feb	6
2016	Feb	7
2016	Mar	9
2016	Mar	11
2016	Apr	14
2016	Apr	15
2016	May	19
2016	May	21

Season 2016-2017

Year	Month	Week	Date
2016	Nov	45	7.11.16
2016	Nov	45	11.11.16
2016	Nov	46	14.11.16
2016	Nov	47	22.11.16
2016	Nov	47	24.11.16
2016	Nov	48	28.11.16
2016	Dec	48	2.12.16
2016	Dec	49	7.12.16
2016	Dec	50	14.12.16
2017	Jan	1	05.01.17
2017	Jan	3	16.01.17
2017	Jan	3	19.01.17
2017	Jan	4	24.01.17
2017	Feb	8	22.02.17
2017	Feb	8	24.02.17
2017	Mar	9	02.03.17
2017	Mar	10	06.03.17
2017	Mar	10	09.03.17
2017	Mar	11	17.03.17
2017	Mar	12	23.03.17
2017	Mar	13	30.03.17
2017	Apr	15	11.04.17
2017	Apr	15	14.04.17
2017	Apr	16	20.04.17
2017	Apr	17	25.04.17
2017	May	18	02.05.17
2017	May	18	04.05.17
2017	May	19	08.05.17
2017	May	20	15.05.17
2017	June	22	03.06.17

Season 2017-2018

Year	Month	Week	Date
2017	Oct	42	17.10.17
2017	Dec	51	19.12.17
2018	Mar	11	12.03.18
2018	May	18	02.05.18
2018	July	30	24.07.18

Color Code	
(red)	Summer
(grey)	Winter
(light blue)	Early Spring
(orange)	Late Spring
(green)	Summer

Supplementary figure 1b.

Season 2016-2017

Year	Month	Week	Date
2017	Mar	9	02.03.17
2017	Mar	10	06.03.17
2017	Mar	10	09.03.17
2017	May	20	15.05.17
2017	June	22	03.06.17
2018	July	30	24.07.18

Season 2017-2018

Year	Month	Week	Date
2018	July	30	24.07.18

Supplementary figure 1c.

Season 2016-2017

Year	Month	Week	Date
2016	Nov	45	11.11.16
2016	Nov	46	14.11.16
2016	Dec	48	2.12.16
2016	Dec	50	14.12.16
2017	Jan	1	05.01.17
2017	Feb	8	24.02.17
2017	Mar	9	02.03.17
2017	Mar	10	06.03.17
2017	Mar	10	09.03.17
2017	Apr	15	14.04.17
2017	Apr	16	20.04.17
2017	Apr	17	25.04.17
2017	May	18	02.05.17
2017	May	20	15.05.17
2017	June	22	03.06.17

Season 2017-2018

Year	Month	Week	Date
2017	Oct	42	17.10.17
2017	Dec	51	19.12.17
2018	Mar	11	12.03.18
2018	May	18	02.05.18
2018	July	30	24.07.18

Supplementary figure 1d.

Season 2016-2017

Year	Month	Week	Date
2017	Mar	9	02.03.17
2017	Mar	10	06.03.17
2017	Mar	10	09.03.17
2017	May	20	15.05.17
2017	June	22	03.06.17
2018	July	30	24.07.18

Season 2017-2018

Year	Month	Week	Date
2018	July	30	24.07.18

Supplementary figure 1. Sampling for seasonal profiling ǁ 1a. Sampling dates for Fluorescence (2015-2016, 2016-2017 and 2017-2018) and P700 measurements (only 2016-2017 and 2017-2018). **1b.** Sampling dates for Time resolved measurements (2016-2017 and 2017-2018). **1c.** Sampling dates for seasonal electron Microscopy (2016-2017 and 2017-2018). 1d. Sampling dates for protein quantification (2016-2017 and 2017-2018).

Supplementary figure 2.

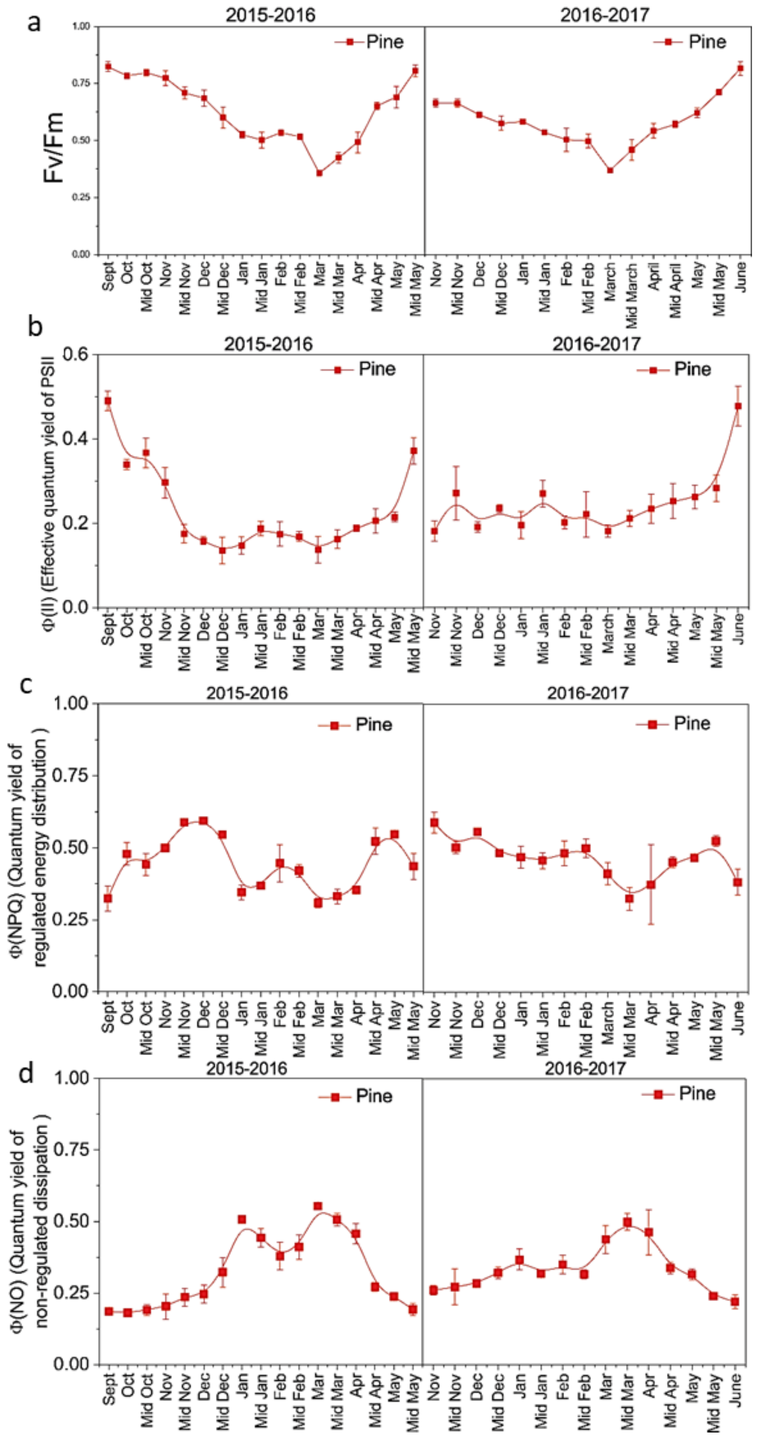

Supplementary figure 2. Seasonal performance of PSII during 2015-2016 (Left panel) and 2016-2017 (Right panel) ‖ a. Changes in maximal quantum efficiency of PSII measured as Fv/Fm. **b.** Effective quantum yield of PSII [Φ(II)]**. c.** Energy dissipation measured as regulated non photochemical quenching [Φ(NPQ)]. **d.** Energy dissipation measured as non-regulated non photochemical quenching[Φ(NO)]. Quantum yields were calculated at actinic light illumination of 300 µmol m^{-2} s^{-1} in the light response curves. All measurements were taken after 30 min of dark adaptation at 4°C in winter and room temperature in summer. All data are means ± SD (n = 3).

Supplementary figure 3.

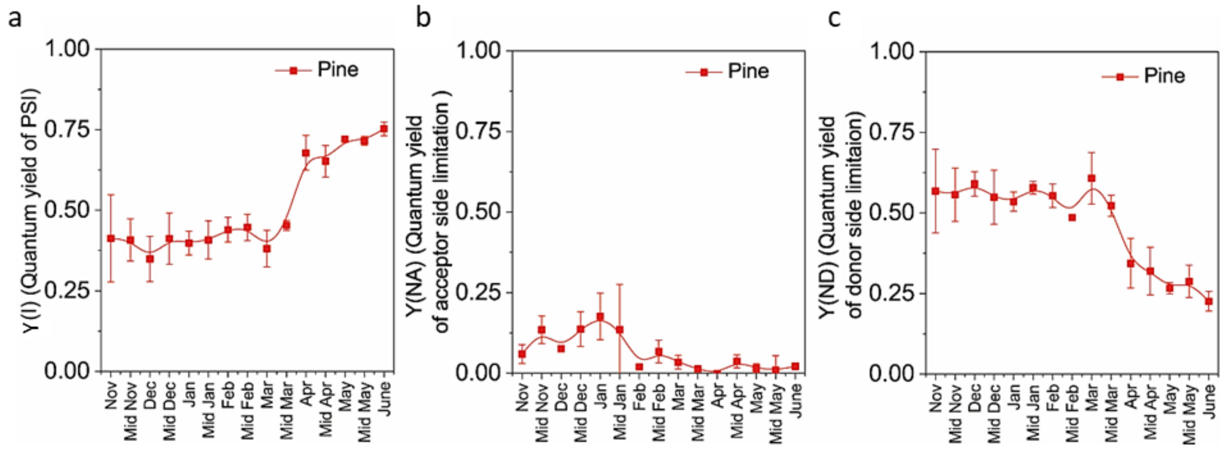

Supplementary figure 3. **Seasonal performance of PSI during 2016-2017 ǁ** Energy distribution in PSI considering Y(I)+Y(ND)+Y(NA) = 1, where Y(I) [Φ(I)], Y(NA) and Y(ND) are **(a)** photochemical quantum yield of PSI (when P700 is reduced and A is oxidised), **(b)** energy dissipation in PSI (measure of acceptor side limitation, when P700 and A both are reduced) and **(c)** energy dissipation in PSI (measure of donor side limitation, when P700 and A both are oxidised), respectively. Quantum yields were calculated from 300 µmol m^{-2} s^{-1} light illumination period of a light response curve. All measurements were taken after 30 min of dark adaptation at 4°C in winter and room temperature in summer. All data are means ± SD (n = 3).

Supplementary figure 4.

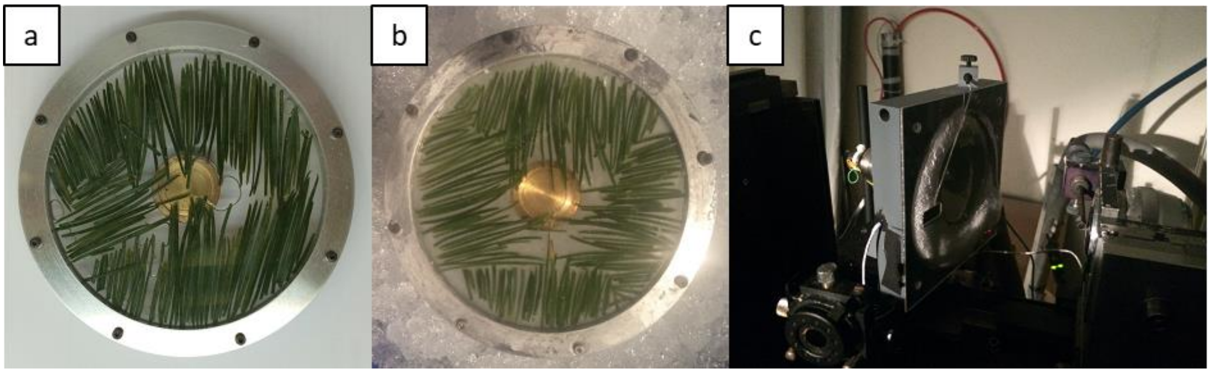

Supplementary figure 4 Lifetime measurements of pine needles ǁ Measuring cuvette with pine needles inside in Summer [S] state **(a),** or E.spring [ES] state **(b)**. **c**. Temperature control chamber, with the cuvette inside it during the experiment.

Supplementary figure 5.

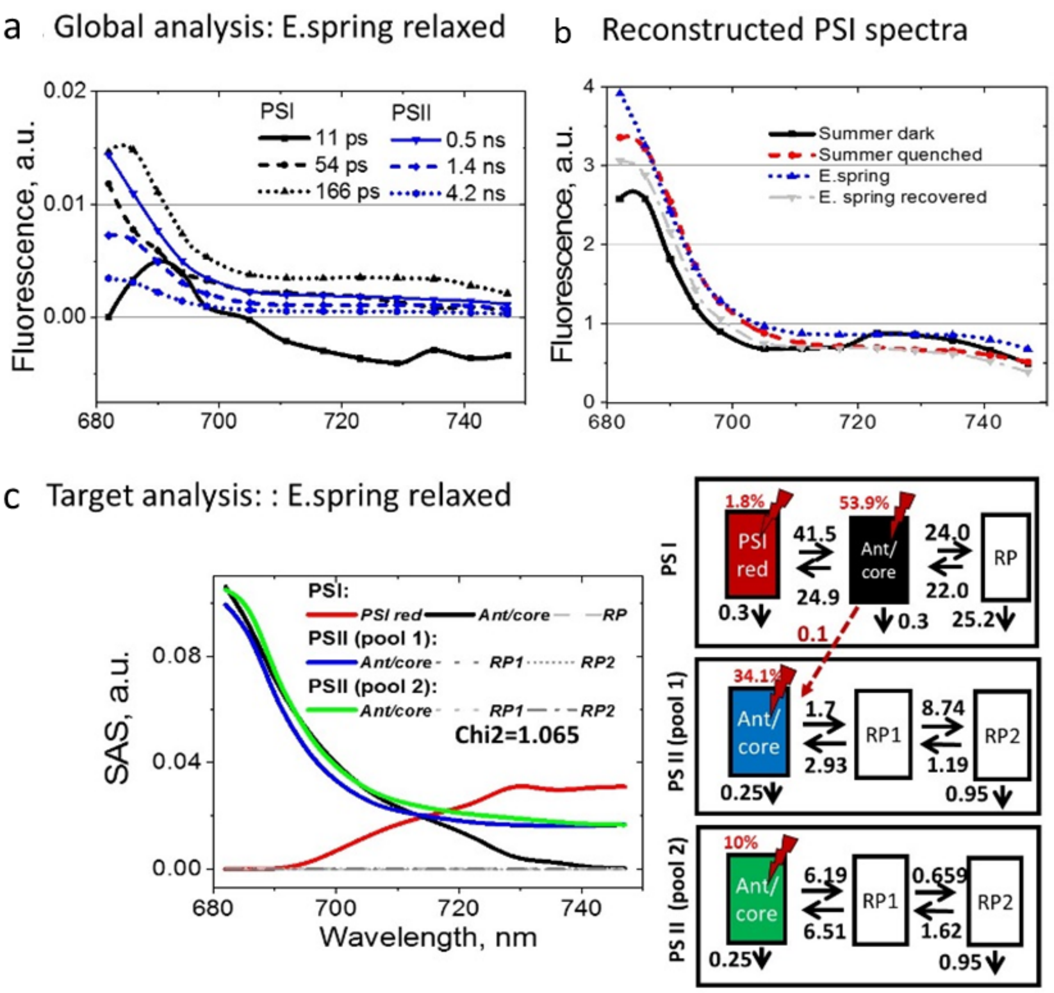

Supplementary figure 5. Lifetime measurements of pine needles ‖ (a) Global analysis and **(c)** target analysis of E.spring needles recovered for 48 h (ER state). The kinetic target analysis (SAS left, kinetic model with rate constants in ns^{-1}, right) shows the results of the detailed target modeling of the fluorescence kinetics of pine needles. The rate constants (ns^{-1}) and Species-associated emission spectra (SAS) resulted were determined from global target analysis. Species-associated emission spectra (SAS) resulted from the fit of the target kinetic model in the corresponding state.

(b) Reconstructed steady-state PSI spectra in four measured states, i.e., Summer (S), Summer quenched (SQ), E.spring (ES) and E.spring recovered (ER).

Supplementary figure 6.

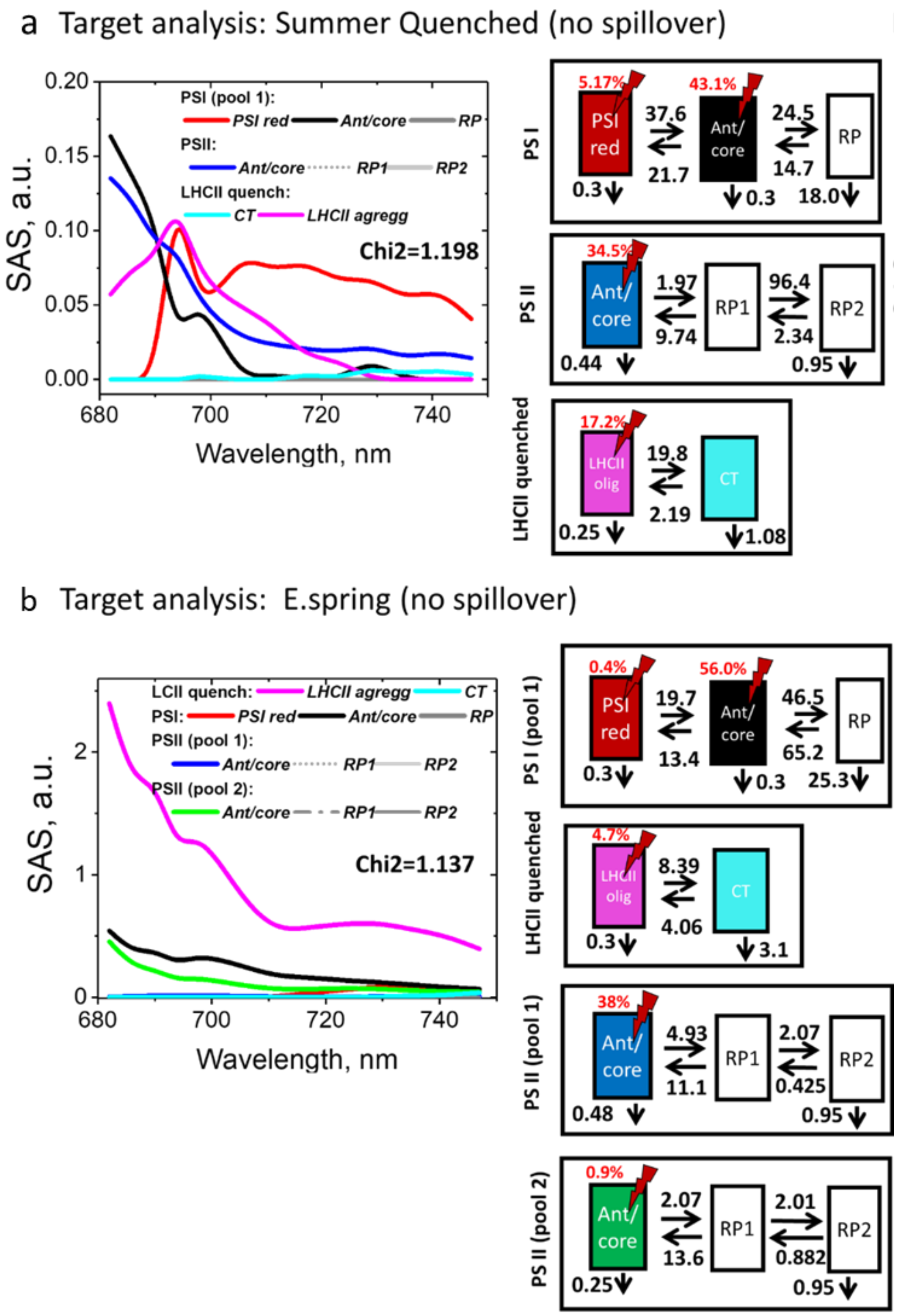

Supplementary figure 6. Lifetime measurements of pine needles | Targeted analysis of fluorescence kinetics of pine needles without spillover mechanism present **(a)** Summer quenched needles (SQ), and **(b)** E.spring needles (ES). The kinetic target analysis (SAS left, kinetic model with rate constants in ns^{-1}, right) shows the results of the detailed target modeling of the fluorescence kinetics of pine needles. The rate constants (ns^{-1}) and Species-associated emission spectra (SAS) resulted were determined from global target analysis. Species-associated emission spectra (SAS) resulted from the fit of the target kinetic model in the corresponding state.

Supplementary figure 7a.

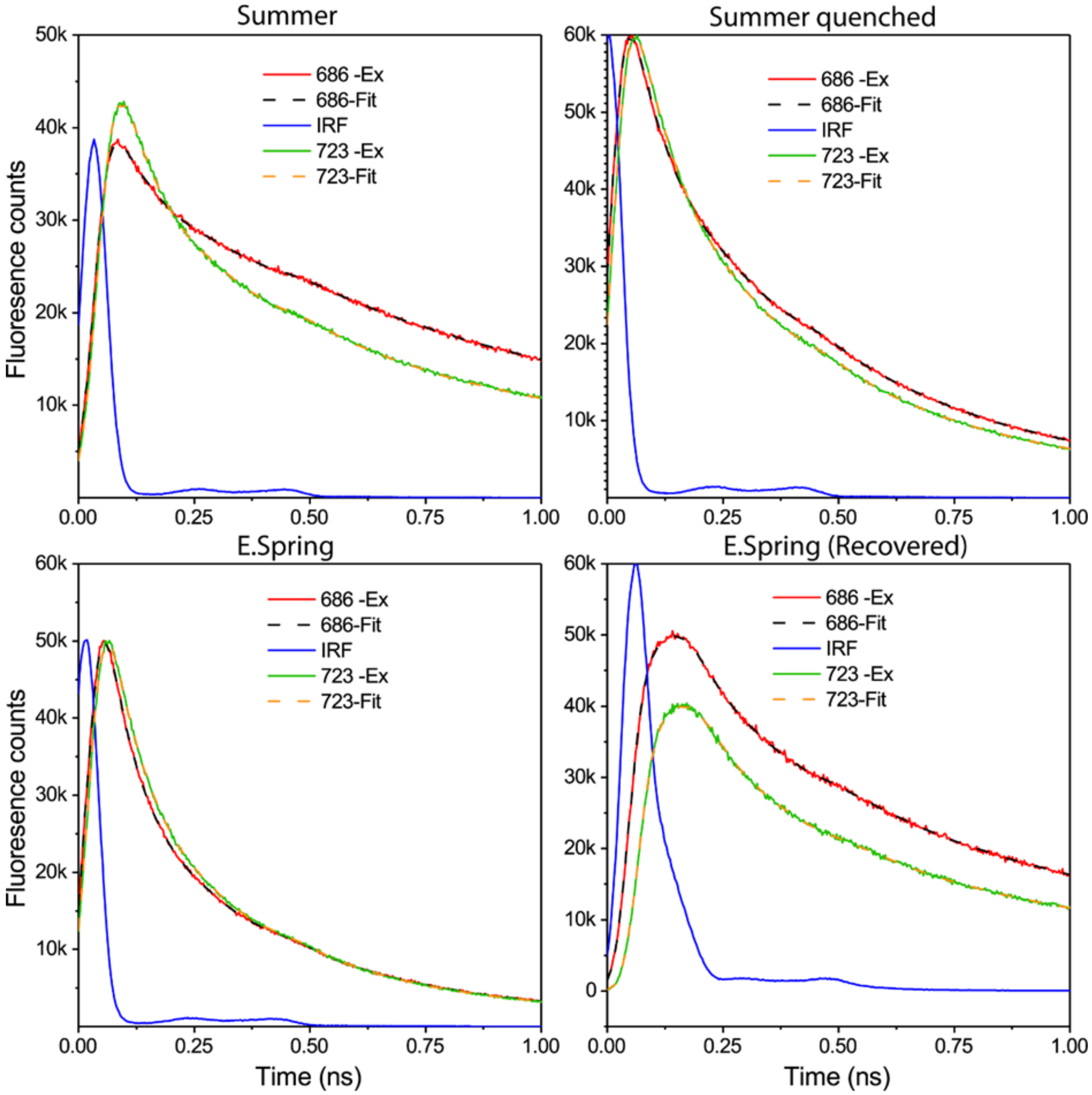

Supplementary figure 7a. Lifetime measurements of pine needles ‖ Example fluorescence traces showing fitting of the data. Both IRF (Instrument response function), experimental data (Continuous line) and fitted data (Dashed line) are shown. Traces are shown from two different wavelengths [686 nm -red (mainly PSII, LHCII contributions) and 723 nm -green (mainly PSI contribution)] as an example.

Supplementary figure 7b.

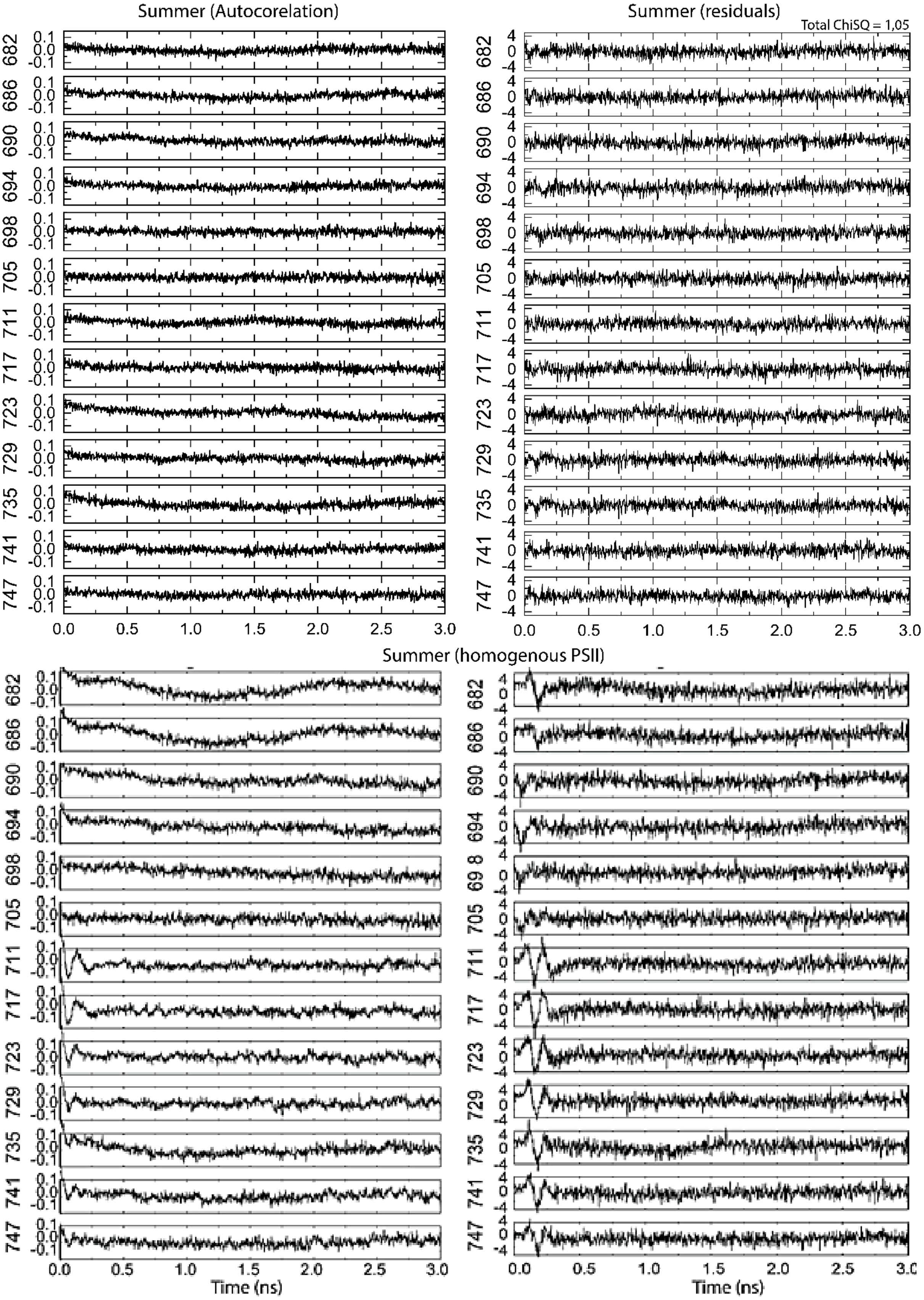

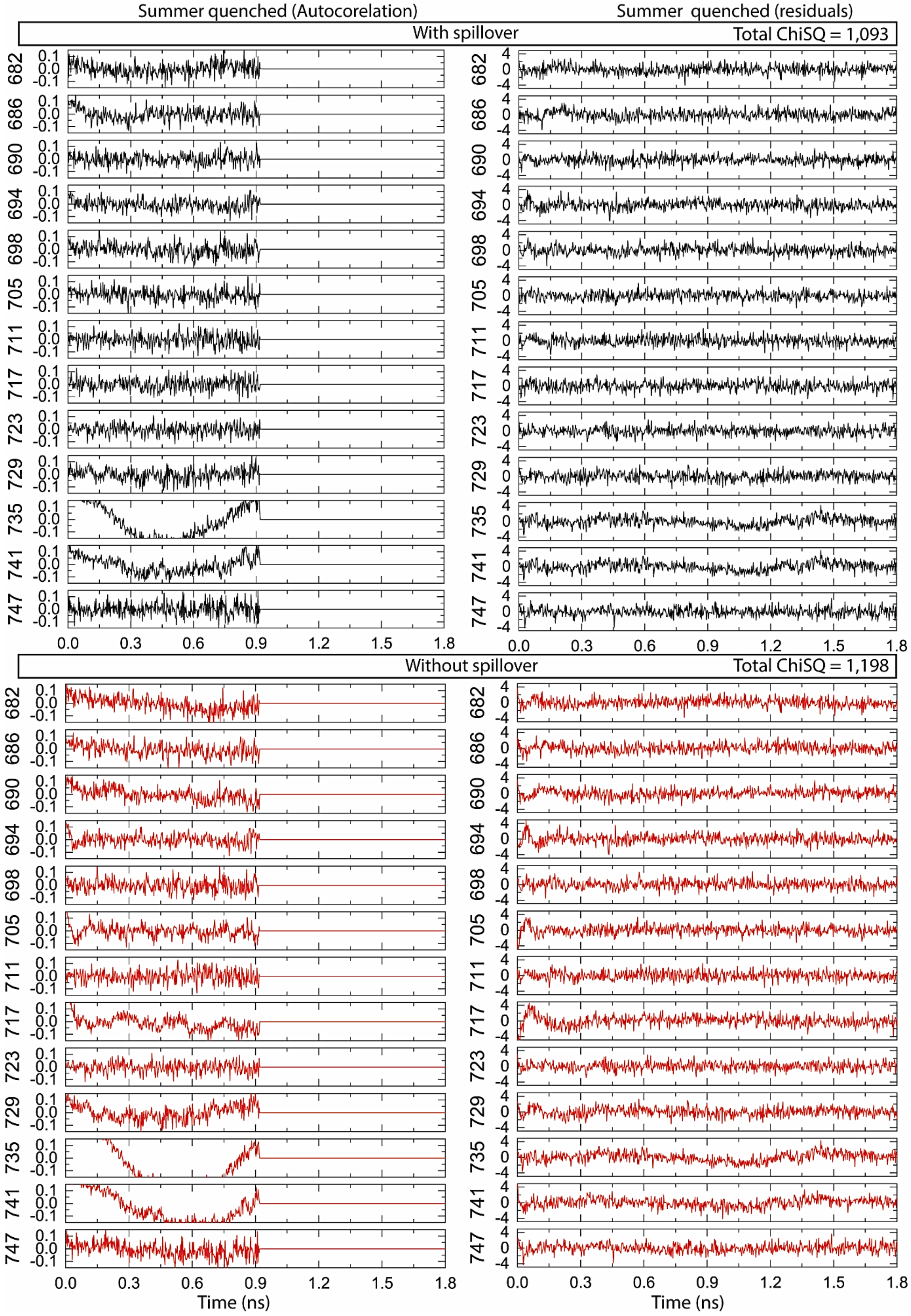

Summer quenched (Autocorelation)
Summer quenched (residuals)
With spillover
Total ChiSQ = 1,093
Without spillover
Total ChiSQ = 1,198
682
686
690
694
698
705
711
717
723
729
735
741
747
0.1
0.0
-0.1
4
0
-4
0.0
0.3
0.6
0.9
1.2
1.5
1.8
Time (ns)

Supplementary figure 7c.

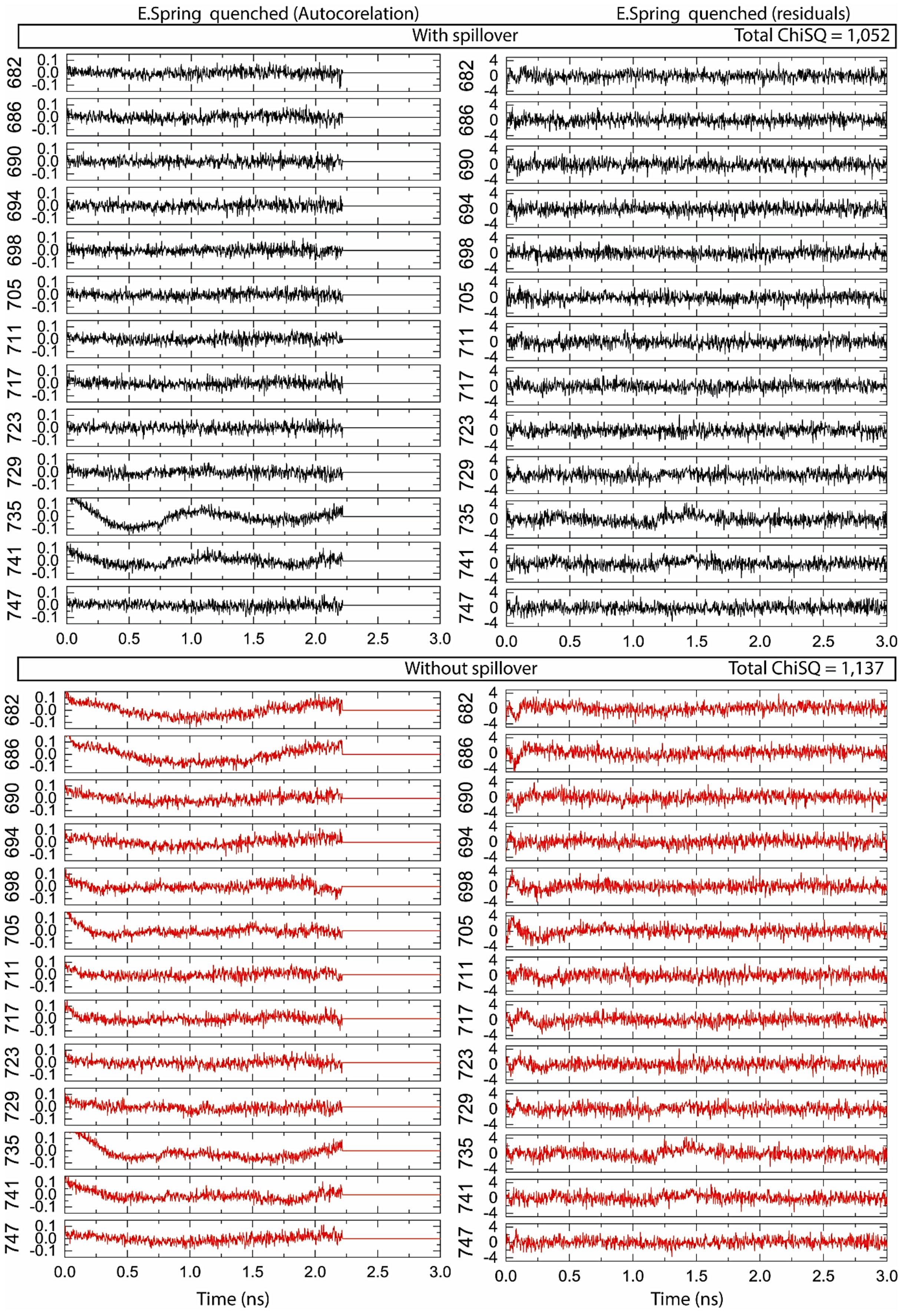

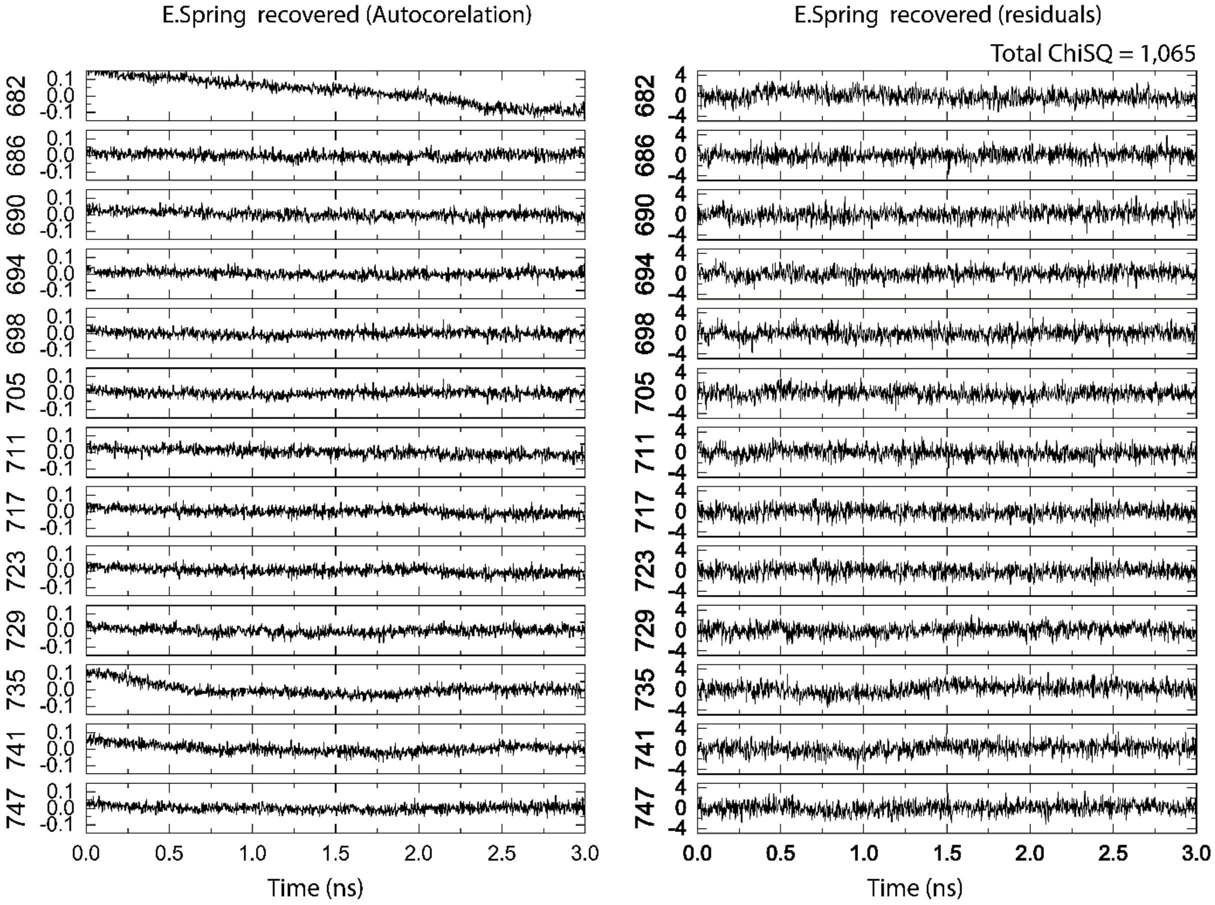

Supplementary figure 7b/c. Lifetime measurements of pine needles ‖ b. Autocorelation and residual plot [Summer (S) and Summer quenched (SQ)]. **c.** Autocorelation and residual plot [E.spring (ES) and E.spring recovered (ER)].

Supplementary figure 8.

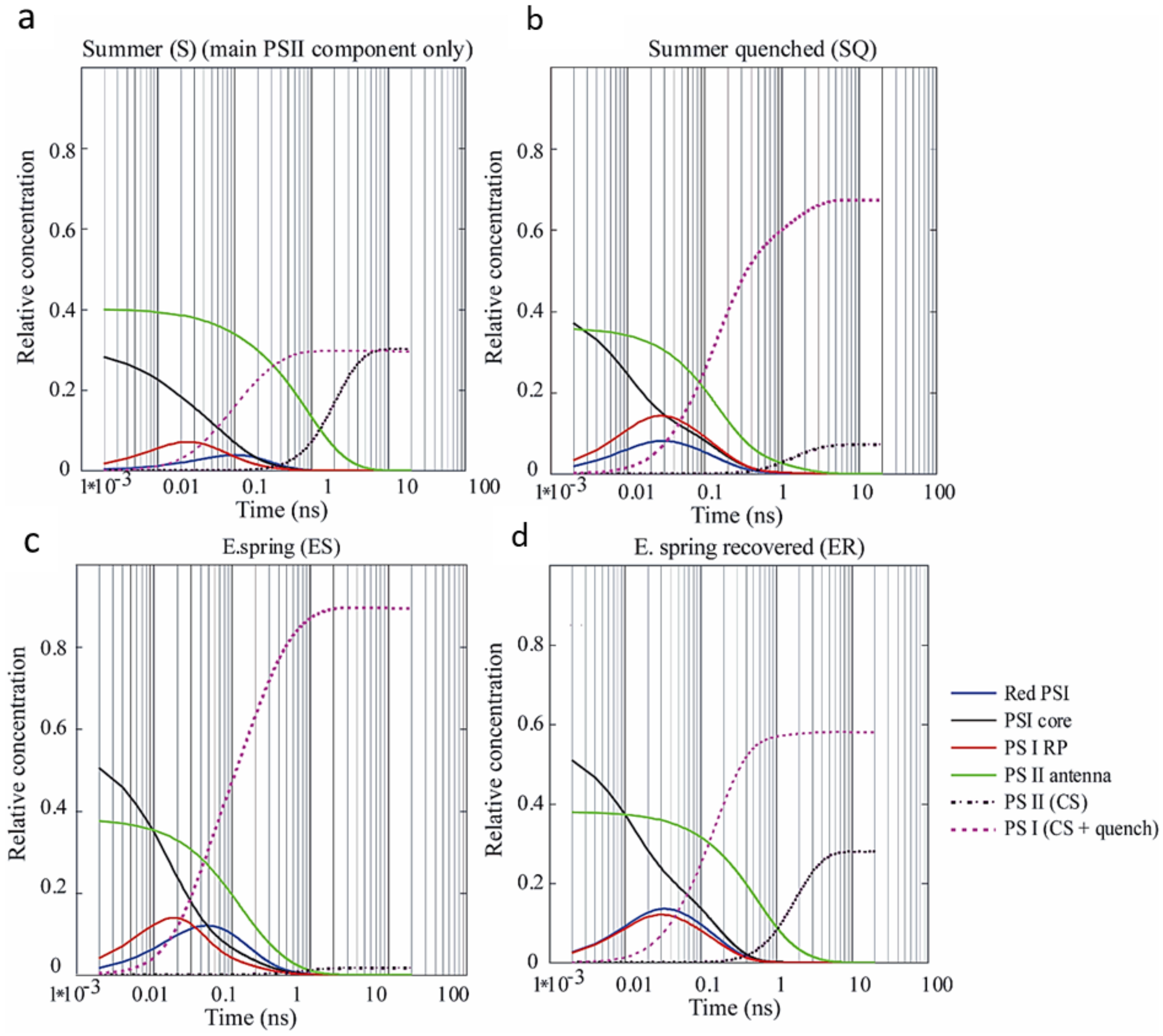

Supplementary figure 8. Time-dependent (on log time scale) populations of selected PSII and PSI compartments as calculated from the fluorescence kinetics (Fig. 3c) ‖ The concentration populations were obtained by resolving the system of differential equations describing PSI or PSII from target model against time. The dashed/dotted curves show the kinetics energy (normalized to the total absorption cross-section) flowing into PSI (purple dashed curves) and PSII (dotted black curves). The initial excitation input was taken from the excitation vectors of corresponding target analysis results (Fig 3c). Depending on the state of the respective reaction center, that energy will be either used for photochemistry or will be deactivated non-radiatively (quenching). See Table 4 SI for the percentages. Black (PSI) and green (PSII) curves show the time course of the excited state populations of the PSs.

Supplementary figure 9.

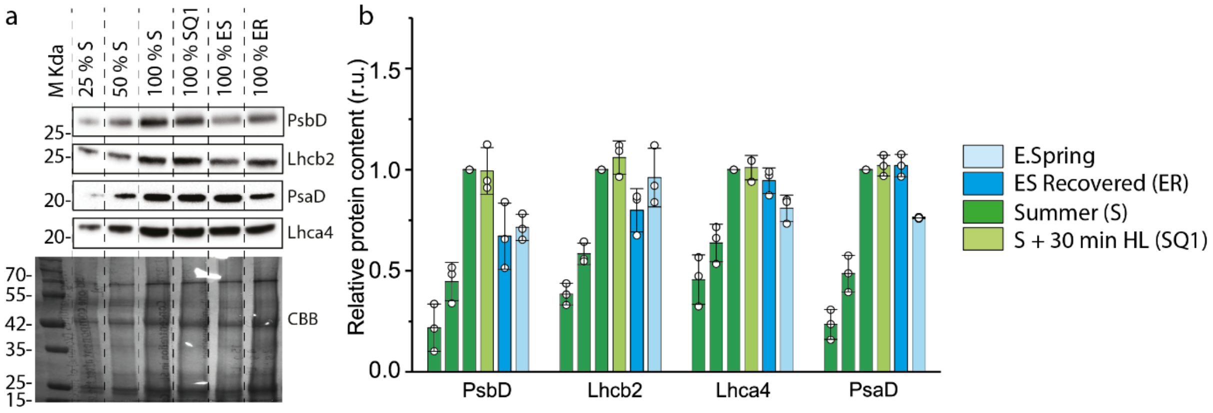

Supplementary figure 9. Protein composition of pine needles collected during different measuring states ‖ a. SDS_PAGE separation of thylakoid proteins loaded based on equal chlorophyll. **b.** Quantification protein by specific antibodies against PsbD, Lhcb2, PsaD and Lhca4, all protein levels were normalized to summer (S) values for each individual replicates. All data are means ± SD (n = 3).

Supplementary Tables.

Supplementary table 1

Parameters	Autumn (A)	Winter (W)	E.Spring (ES)	L.Spring (LS)	Summer (S)
**Number of chloroplasts**	15.2±3.93a	12.73±3.72c	13.46±4.05a	14.2±4.26	16.3±2.38
**Number of grana per chloroplast**	23.07±6.9c	18.66±9.24c	18.73±9.88c	25.38±9.88	27.47±8.62
**Number of thylakoids per granum**	4.97±0.27c	4.02±0.34c	2.72±0.46c	2.85±0.51c	6.50±0.33
**Lipid globules per chloroplast**	27.3±19.81c	50.37±16.23c	55.7±15.07c	33.125±18.00c	15.67±6.29

Quantitative analysis of seasonal changes in chloroplast ultrastructure as seen in Transmission electron microscopy. Statistical significance levels are referred as a, b, c denoting 99.95%, 99.99% and 99.999% confidence level.

Supplementary table 2

	**Summer (S)**	**Summer Quenched (SQ)**	**E.spring recovered (ER)**	**E. spring (ES)**
Chl a/b	2.85±0.15	2.83±0.14	2.54±0.16	3.36±0.20
Chl /Car	4.90±0.48	4.67±0.34	2.72±0.10	2.98±0.45
Chl/fr w, mg/g	1.06±0.17	1.05±0.31	0.64±0.26	0.55±0.05
Carotenoids/ Chl a				
neo	0.23±0.02	0.23±0.02	0.52±0.08	0.37±0.28
vio	0.26±0.03	0.29±0.06	0.77±0.22	0.15±0.03
lut	0.83±0.21	0.96±0.27	2.61±0.14	2.15±0.43
beta	0.27±0.09	0.37±0.12	0.16±0.003	0.48±0.09
zea	n.d.	n.d.	n.d.	0.58±0.11

Pigment composition analysis by HPLC. Chl, Chlorophyll; fr w, fresh weight; neo, neoxanthin; vio, violaxanthin; lut, lutein; beta, beta-carotene; zea, zeaxanthin. Shown is ±SD (n=3).

Supplementary table 3

< τ >, ps		Summer (S)	Summer Quenched (SQ)	E.spring recovered (ER)	E. spring (ES)
PSI		95	95	90	42
PSII	pool 1	988	357	820	228
	pool 2	1228		2113	2950
	total	1086		1137	273
LHCII quenched			399		420
total		779	296	572	170

To assess differences in excited-state energy relaxation of different decaying components we calculated the average excited state relaxation time as $< \tau >= \sum A_i \, \tau_i$, where A_i are the relative areas of each Decay-associated spectra (DAS). DAS were obtained from global target analysis (Fig. 3).

Supplementary table 4

Sample condition	PSI (CS+ quench)	PSII (CS)	Comments
Summer (S)	30%	50%	Fig. 4.3 SI shows only the main PSII pool contribution but two PSII pools were used in the calculation
Summer quenched (SQ)	67%	7.1%	Detached LHCII quenched was not considered
E.Spring recovered (ER)	55%	27%	small amount of photoinhibited PSII pool (pool 2) was not considered
E.Spring (ES)	89%	1.5%	unquenched PSII (pool 2) and detached LHCII quenched were not considered.

Percentages of total energy flow into PSII and PSI (corresponding to the components PSII (CS) and PSI (CS+quench), respectively) as deduced from Fig. 4.3 SI. The actual energy use (charge separation (CS) or quenching) depends on the condition of the respective reaction center, either open or closed, at the given condition.
