# Supplementary material for: Direct energy transfer from photosystem II to photosystem I confers winter sustainability in Scots Pine
Source: Nat Commun. 2020 Dec 15;11:6388. doi: 10.1038/s41467-020-20137-9 (PMC7738668; doi:10.1038/s41467-020-20137-9)
Supplement: Supplementary file 3 — Source Data [file 41467_2020_20137_MOESM3_ESM.zip › Source data file_Bag et al., 2020/Source data file/Blot images Supplementary information 5-I/All data labelled.pptx]

## Slide 1
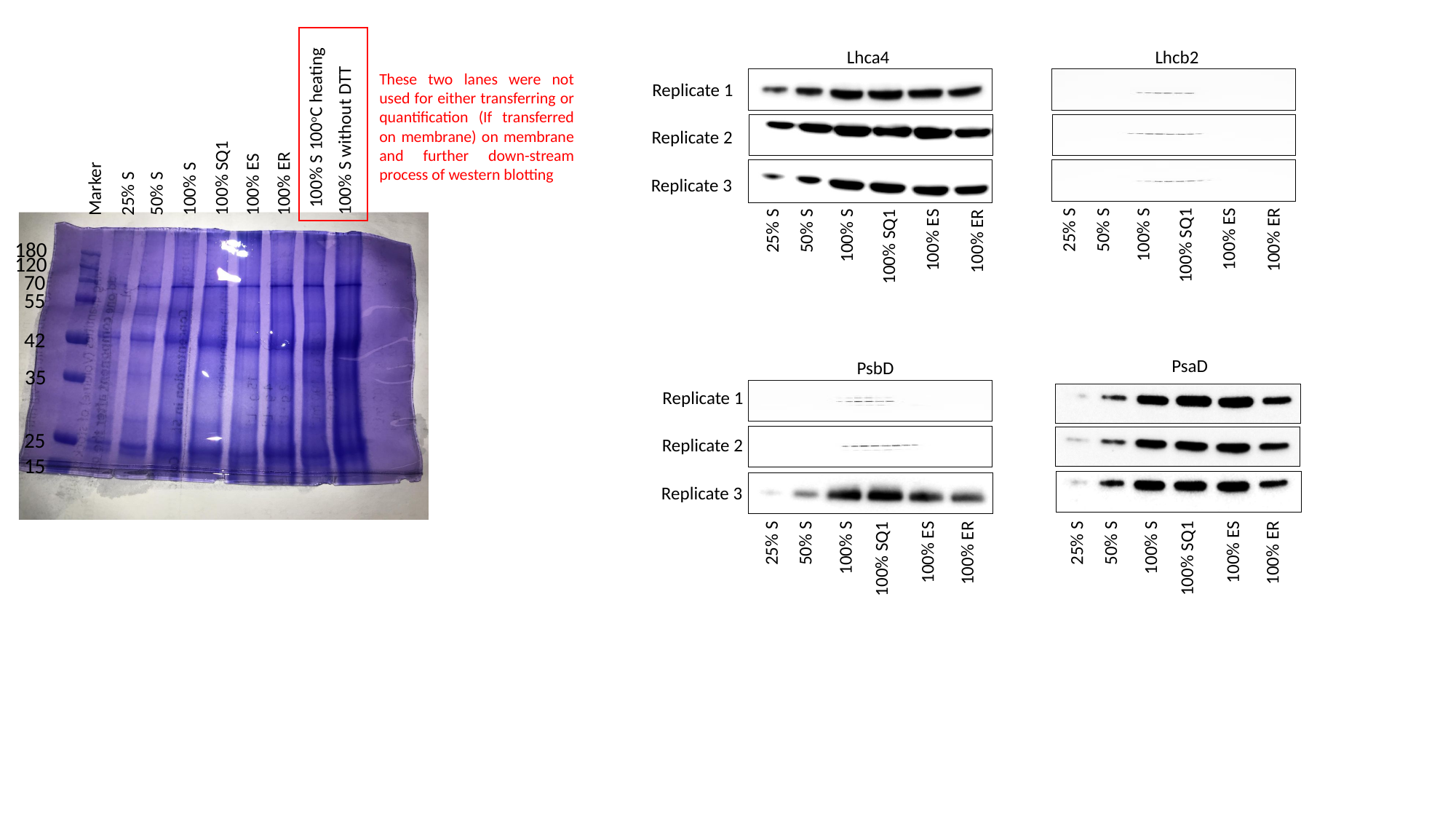

Lhcb2
Lhca4
These two lanes were not used for either transferring or quantification (If transferred on membrane) on membrane and further down-stream process of western blotting
Replicate 1
100% S 100oC heating
Replicate 2
100% S without DTT
100% SQ1
100% ER
100% ES
Replicate 3
Marker
100% S
25% S
50% S
25% S
50% S
25% S
50% S
100% S
100% S
100% ES
100% ER
100% ES
100% ER
100% SQ1
100% SQ1
180
120
70
55
42
PsaD
PsbD
35
Replicate 1
25
Replicate 2
15
Replicate 3
25% S
50% S
25% S
50% S
100% S
100% S
100% ES
100% ES
100% ER
100% ER
100% SQ1
100% SQ1
