# Supplementary figures and images for: Direct energy transfer from photosystem II to photosystem I confers winter sustainability in Scots Pine
Source: Nat Commun. 2020 Dec 15;11:6388. doi: 10.1038/s41467-020-20137-9 (PMC7738668; doi:10.1038/s41467-020-20137-9)

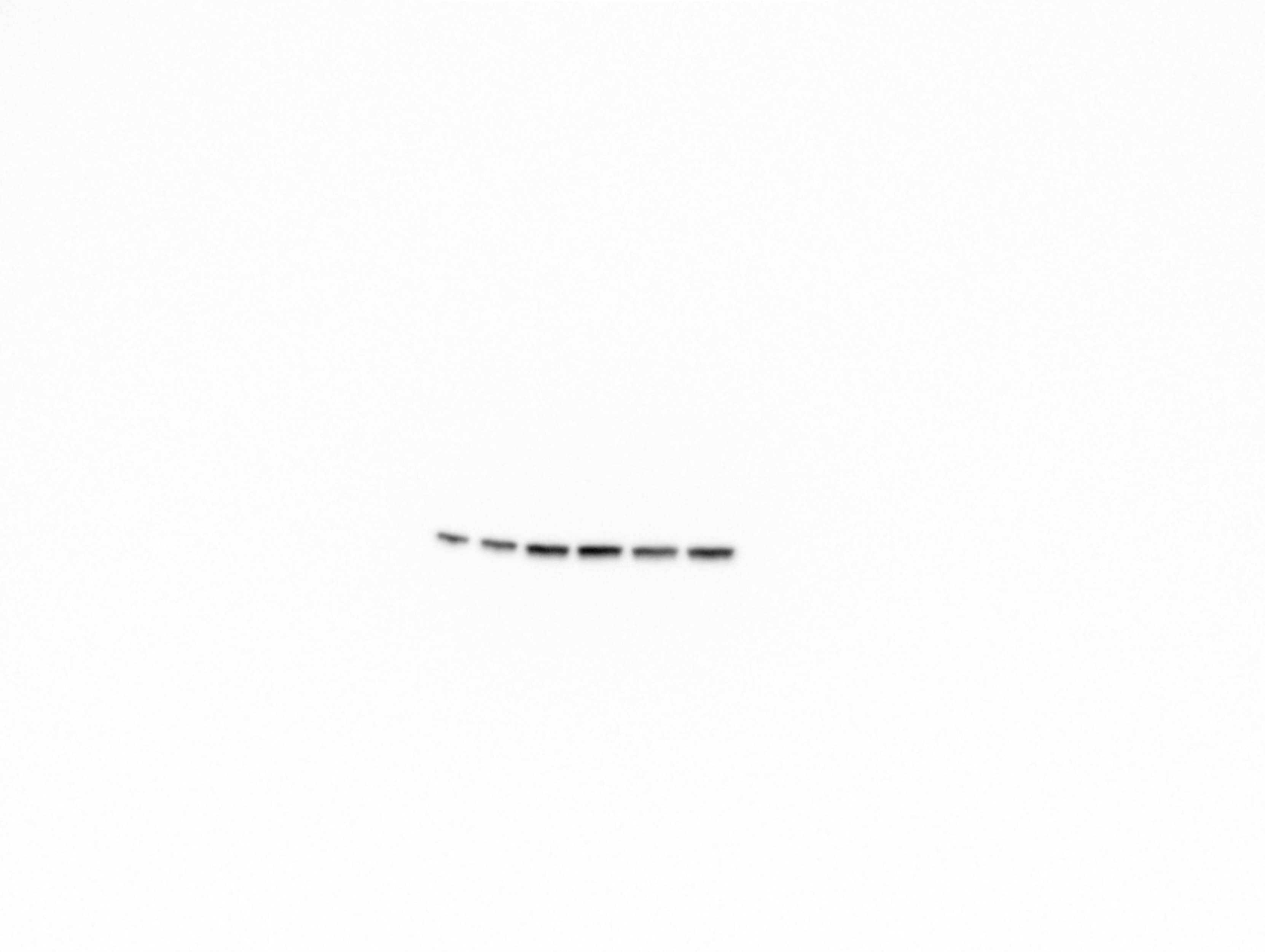

Supplement: Supplementary file 3 — Source Data [file 41467_2020_20137_MOESM3_ESM.zip › Source data file_Bag et al., 2020/Source data file/Blot images Supplementary information 5-I/LHCB2.tif]

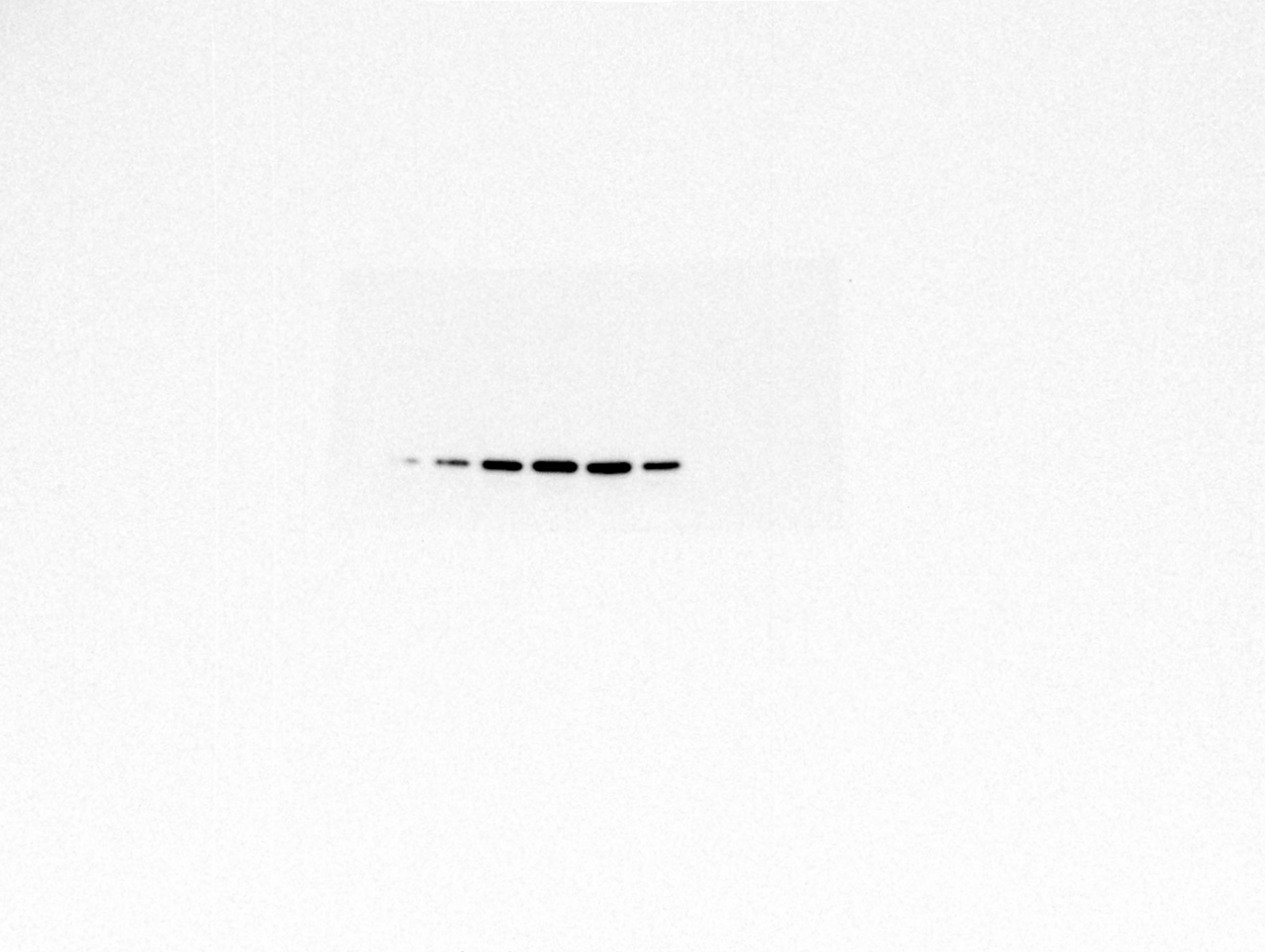

Supplement: Supplementary file 3 — Source Data [file 41467_2020_20137_MOESM3_ESM.zip › Source data file_Bag et al., 2020/Source data file/Blot images Supplementary information 5-I/PSAD.tif]

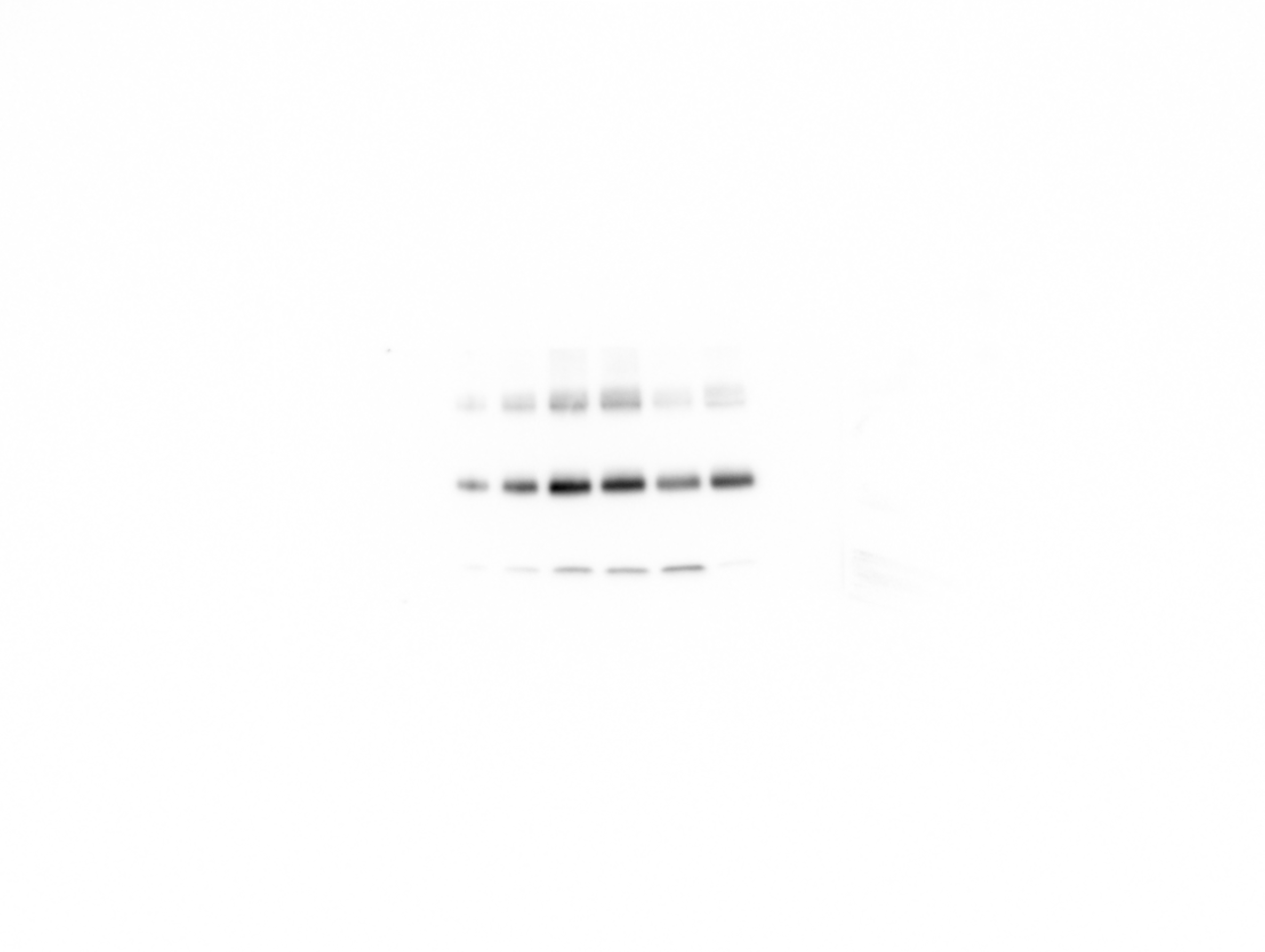

Supplement: Supplementary file 3 — Source Data [file 41467_2020_20137_MOESM3_ESM.zip › Source data file_Bag et al., 2020/Source data file/Blot images Supplementary information 5-I/PSBD.tif]

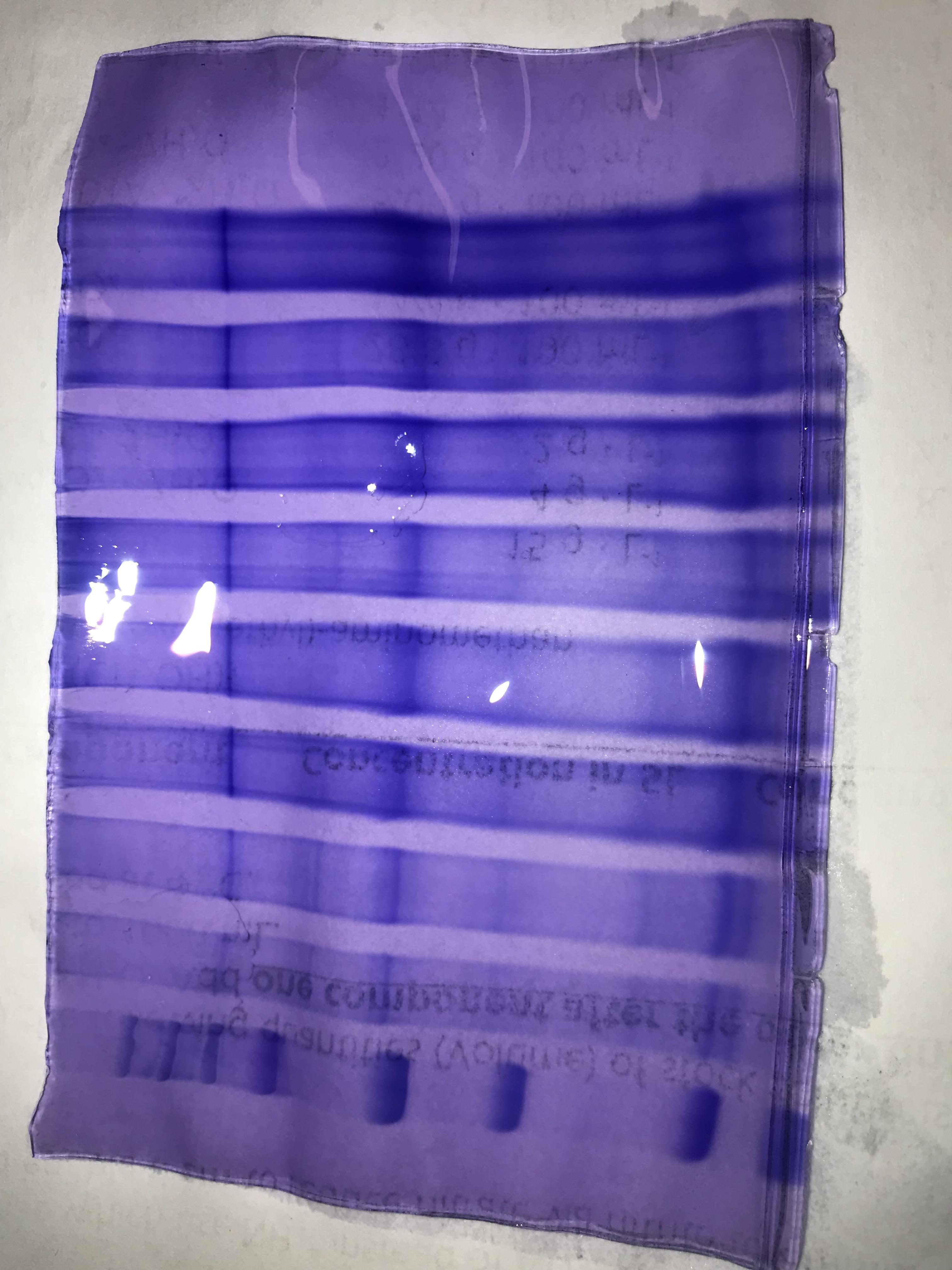

Supplement: Supplementary file 3 — Source Data [file 41467_2020_20137_MOESM3_ESM.zip › Source data file_Bag et al., 2020/Source data file/Blot images Supplementary information 5-I/Full gel scan_CBB.jpg]

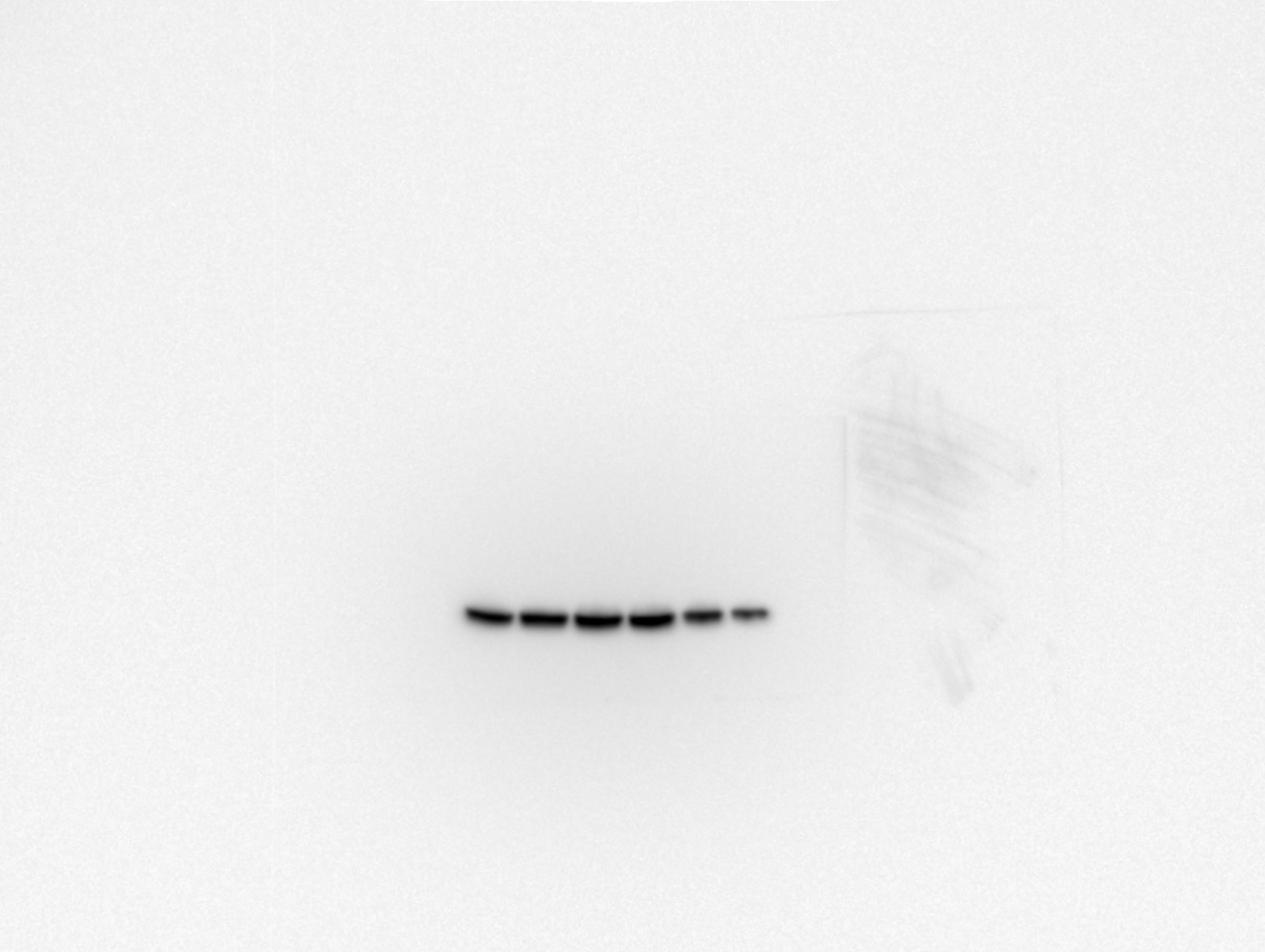

Supplement: Supplementary file 3 — Source Data [file 41467_2020_20137_MOESM3_ESM.zip › Source data file_Bag et al., 2020/Source data file/Blot images Supplementary information 5-I/LHCA4.tif]
